# Distribution of Essential and Toxic Elements in *Pelecus cultratus* Tissues and Risk Assessment for Consumer Health

**DOI:** 10.3390/toxics11080715

**Published:** 2023-08-20

**Authors:** Aleksandra Aleksandrovna Payuta, Ekaterina Aleksandrovna Flerova, Yulia Vladimirovna Zaitseva

**Affiliations:** Scientific Laboratory Ecobiomonitoring and Quality Control, P.G. Demidov Yaroslavl State University, 150003 Yaroslavl, Russia; a.payuta@mail.ru (A.A.P.); katarinum@mail.ru (E.A.F.)

**Keywords:** sabrefish *Pelecus cultratus*, liver, gonads, heavy metals, Rybinsk Reservoir, risk assessment

## Abstract

Nowadays, the problem of inland water pollution is acute. It is caused by vast industrial growth and agricultural intensification. Concentrations of Cd, Pb, Zn, Cu, Mn, Fe, Mg, and Kwere determined in the muscles, liver, and gonads sabrefish from Rybinsk Reservoir areas with different anthropogenic loads. The tissue samples were analyzed by atomic absorption spectrometry. Heavy metals accumulated more intensively in the body of fish from more polluted areas of the reservoir. Among the analyzed elements, the maximum accumulation levels were found for K, Zn, and Fe and the minimum levels were observed for Cd and Pb. The gonads contained the largest concentration of Cd and Mn, the muscles contained the highest concentrations of Mg, and the other elements mainly accumulated in the liver of sabrefish. The THQ and HI values for all elements did not exceed 1, which suggests that there is no potential non-carcinogenic risk to human health. The target values of carcinogenic risk (TR) for cadmium ranged from 8.32 × 10^−6^ to 1.22 × 10^−4^ in the muscles. The increased content of cadmium in the gonads of sabrefish not only poses a risk to human health, but also to the reproduction of this species in the Rybinsk Reservoir.

## 1. Introduction

Nowadays, the problem of inland water pollution is acute. It is caused by vast industrial growth, agricultural intensification, and urban development along the banks of rivers [1,2]. Sources of pollution are often domestic and industrial wastewater, runoff from landfills, agricultural land, and urban areas [3,4,5,6].

Heavy metals are the most ubiquitous environmental pollutants affecting the quality of water resources even in the most remote places on Earth, for example, Tibet, Sundarbans, Amazonia, the Pacific region, the Polar region, and others [7,8,9,10,11,12]. Inland waters such as rivers, lakes, streams, and even groundwater are also subject to heavy metal pollution [13,14,15,16,17,18,19].

The content of pollutants in water reflects only short-term exposure and does not always show the state of biota due to the diffusion of contaminants and their concentrations below the limit of detection, which may increase over time [2,20]. Heavy metals can enter and accumulate in the fish body by chemisorption, mechanical capture of suspended particles, and absorption by gills and through the digestive tract viafood [21]. The latter way is considered the most dangerous because the toxic properties of substances can manifest themselves not only in prey, but also in predators through food webs [22]. Once in the fish body, heavy metals replace important minerals for vital activity and block biological functions, affecting physiological and biochemical parameters [23,24,25]. Therefore, fish are a convenient test object for studying water pollution [26,27,28,29].

Since fish area source of valuable proteins, a number of vitamins, minerals, and fatty acids, especially omega-3, which are essential for human health, they play an important role in the human diet [30,31]. However, after accumulating heavy metals, fish transmit them to humans when eaten [32,33].

Sabrefish *Pelecus cultratus* is a pelagic species widespread in both fresh and brackish waters of Europe and Russia [34,35,36,37,38,39,40]. The species is a planktophage and ichthyophage, able to consume a very wide range of food organisms. Juveniles feed mainly on plankton, while the food of adults is diverse. Both plankton and benthic larvae of chironomids and terrestrial and aquatic insects are found in its intestines. A significant part of the sabrefish diet is juveniles of other fish species [41,42,43]. Due to its broad dietary spectrum, the species is characterized by a high nutritional value [44,45]. Sabrefish is a target for commercial fisheries, which in a number of countries has led to the threat of its extinction [46,47,48].

There are only a few studies on the elemental composition of this fish species. Attention has mainly been paid to studies of the mercury content in sabrefish meat [49,50,51]. A number of works are devoted to the study of heavy metal concentrations in the muscles of commercial fish species, including sabrefish [52,53,54]. The most comprehensive study, which includes analysis of different tissues of this species, was conducted in the Danube River [55]. We have not found any works on the elemental composition of various tissues of sabrefish from water bodies in Russia or health risk assessments associated with the consumption of this species by humans.

Therefore, studies to determine the elemental composition of sichel tissues are relevant for ecological monitoring of water bodies, as well as for assessing both the physiological state of individuals and the quality of fish products consumed by humans.

The purpose of the work is to study the features of the accumulation of essential and toxic elements in the muscles, liver, and gonads of *Pelecus cultratus* and to assess the risks to human health when consuming this species.

## 2. Materials and Methods

### 2.1. Research Area

**Figure 1:**
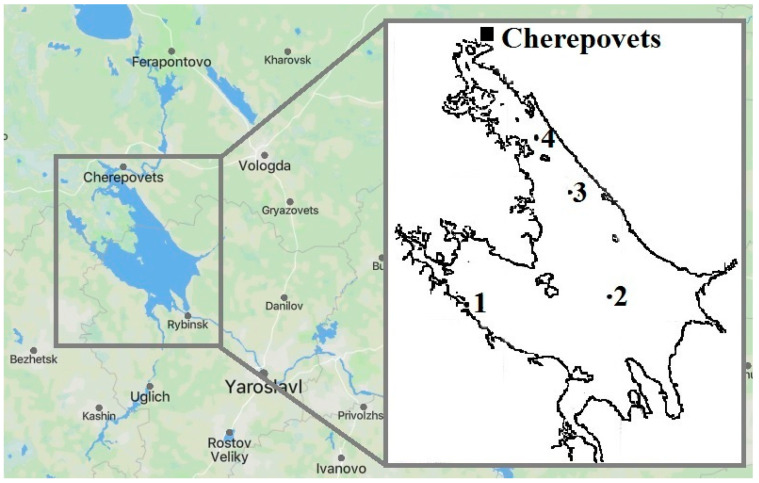
Schematic map of the Rybinsk Reservoir.

Thisresearch was conducted in the Rybinsk Reservoir, the largest artificial water body in the Russian Federation [56,57]. Its area is 4550 km^2^, its maximum length is 250 km, its width is 70 km, its average depth is 5.6 m, and its catchment area is 150,500 km^2^ [58,59]. The reservoir is a lake-type eutrophic water body [60]. The profundal macrozoobenthos is represented by an oligochaete–chironomid complex [61]. Intense fishing is carried out in this reservoir. For many people living on its shores, fishing remains the only means of subsistence [62,63]. In Cherepovets, the north-eastern part of the reservoir, chemical and metallurgical industries are widely developed. The largest mining and metallurgical company “Severstal” is located there. This area has been subjected to long-term pollution by industrial wastewater, characterized by high concentrations of persistent organic pollutants and heavy metals [64,65]. Scientists have recorded a significant content of heavy metals in the water and bottom sediments [64,66]. In addition, the area is exposed to household wastewater, as well as diffuse runoff from agricultural land and highways [67]. The adverse impact of industrial pollution has been described in numerous research papers [68,69,70,71,72,73,74,75]. Sampling sites where sabrefish were caught differ in the level of anthropogenic load (Figure 1).

Station 1 (58°23′ N, 37°45′ E.) is considered conditionally clean and at Station 2 (58°25′ N, 38°29′ E), some increased heavy metal concentrations in bottom sediments have been detected. Stations 3 (58°43′ N, 38°16′ E.) and 4 (58°51′ N, 38°06′ E) have the status of heavily polluted. They are located near the metallurgical industrial complex—the main source of reservoir pollution [64,76,77].

### 2.2. Sampling

All procedures with fish were performed in accordance with the ARRIVE guidelines for the use of animals for research purposes [78].

A total of 39 sabrefish individuals were caught by trawl nets at the end of the feeding period (September–early October) (Table 1). We chose this early fall sampling season to exclude spawning periods, when shifts in biochemical parameters of fish muscles and organs occur.

In order to acclimate after capture, the fish were kept in tanks with river water. After this, each individual fish’s length and weight with and without entrails were measured.

The skin was separated from the skeletal muscles on a refrigerant and tissue samples of the muscle along the spine, as well as of the liver and gonads from the internal cavity, were excised. Prior to analysis, all samples were weighed and frozen at a temperature of −18 °C. Since more males were caught than females (35 vs. 4), the elemental content was analyzed only in the testes.

### 2.3. Heavy Metal Analysis

Concentrations of the following micro- and macro-elements were measured in the fish muscles, liver, and gonads: cadmium (Cd), lead (Pb), zinc, (Zn), copper (Cu), manganese (Mn), iron (Fe), magnesium (Mg), and potassium (K). The tissue samples were analyzed by atomic absorption spectrometry with electrothermal atomization (ETA-AAS) in a KVANT 2-AT spectrophotometer (Kortec Ltd., Moscow, Russia). The ash was dissolved in 5 mL of 20% HCl and filtered through filter paper. The correctness of determining the concentration of elements was checked using the state standard samples for atomic absorption spectrophotometry. The concentration of potassium was determined using the official methods of AOAC International [79]. The results obtained were expressed in mg/kg of wet weight.

### 2.4. Health Risk Assessment

It is known that heavy metals can enter the human body and affect it in three ways: through the gastrointestinal tract or skin or via inhalation [80]. In this study, the first pathway was considered to assess the risk to human health. To determine the risk over a lifetime of fish consumption, the target hazard quotient (THQ), hazard index (HI), and target cancer risk (TR), determined by generally accepted Formulas (1), (2), and (3), respectively, were used [81,82,83].

THQ = EF × ED × Ir × C/RfD × BW × TA,(1)

HI = THQ_Cd_ + THQ_Pb_ + … + THQ_n_ …, (2)

TR = EF × ED × Ir × C × CSF/BW × TA,(3)

where EF—exposure frequency (365 days/year), ED—exposure duration (74 years), Ir—daily consumption of fish (according to FAO data for the Russian Federation in 2020; 0.015 kg/day for pelagic fish), C—metal concentration in fish, mg/kg, RfD—reference peroral dose, mg/kg/day, BW—average human weight (74 kg in the Russian Federation), TA—average exposure time (365 days/year×ED), and CSF—cancer slope factor for carcinogenic metals (mg/kg/day).

The RfD values of Cd, Pb, Zn, Cu, Mn, and Fe are 0.001, 0.0035, 0.3, 0.04, 0.14, and 0.7 mg/kg/day, respectively [80,81,84,85]. The CSF for Cd is 15 mg/kg/day and for Pb it is 0.0085 mg/kg/day [81,86].

### 2.5. Statistical Analysis

The data were checked for normality of the distribution using a Shapiro–Wilk test. Since the data did not follow a Gaussian distribution, the Kruskal–Wallis criterion and the Dunn multiple comparison criterion were applied to compare between different sampling areas and between tissues. The results of the study are presented in the form of mean values and their standard deviations (x ± SD). Differences between the compared parameters were considered statistically significant at *p* < 0.05.

## 3. Results

### 3.1. The Concentration of Micro- and Macro-Elements in Sabrefish from Different Areas of the Reservoir, Characterized by Varying Degrees of Anthropogenic Load

The highest Cd and Pb concentrations were found, respectively, in the gonads and liver of sabrefish from Station 4; for Zn, Cu, and Fe, the highest concentrations were found in the liver of individuals from Station 3; for Mn, the highest concentration was found in the gonads of fish from Station 3; and for Mg and K, the highest concentrations were found in the gonads of sabrefish from Station 1 (Table 2).

The lowest concentrations of Cd, Zn, Mn, and K were found in the muscles of sabrefish at Station 1, those of Pb and Cu were found in the gonads of fish from Station 1, and those of Fe and Mg were found in the gonads of individuals at Station 2 (Table 2).

The contents of Cd, Mg, and K in the muscles of sabrefish from Station 1 were significantly lower than at Stations 4, 3, and 2, respectively (Table 2). In the liver of individuals from Station 3, significantly more Zn and Mn had accumulated than in the liver of sabrefish from Station 4, and higher Cu concentrations were recorded than in fish from Station 1. The Cu content in the gonads of sabrefish from Station 1 was significantly lower than in the gonads of fish from Station 3, and the concentration of Fe was higher than in individuals from Station 2.

Regardless of the sampling area, the following pattern of the intensity of element accumulation in the liver of sabrefish was observed: Cd < Pb < Mg < Mn < Cu < Zn < Fe < K. No such dependence was found in the muscles and gonads; however, there was a tendency toward greater accumulation of K, Zn, and Fe in these tissues, the least accumulation of Cd was in the muscles, and the least accumulation of Pb was in the gonads.

### 3.2. Concentration of Micro- and Macro-Elements in Different Parts of Sabrefish

**Figure 2:**
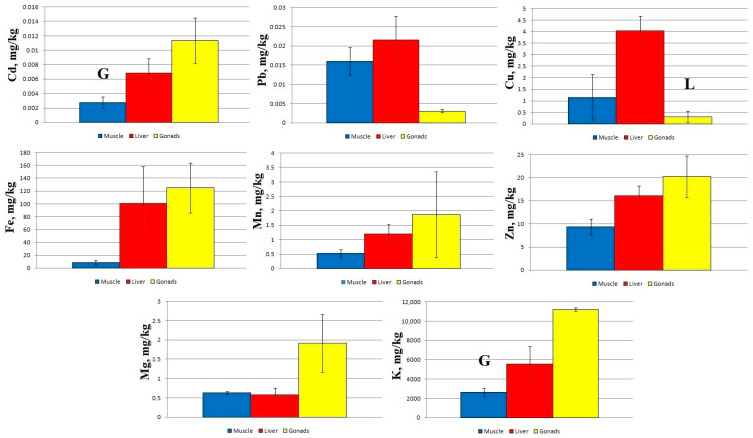
Concentration of micro- and macro-nutrients in different parts of sabrefish from Station 1 of the Rybinsk Reservoir. G—statistically significant differences from the gonads and muscles, respectively. L—statistically significant differences from the liver and gonads, respectively. Muscles *n* = 7, liver *n* = 7, gonads *n* = 6.

A comparison of micro- and macro-nutrient concentrations in different tissues of sabrefish from Station 1 shows significant statistical differences between the concentration of Cd and K in the muscles and gonads, as well as between the concentration of Cu in the liver and gonads (Figure 2).

**Figure 3:**
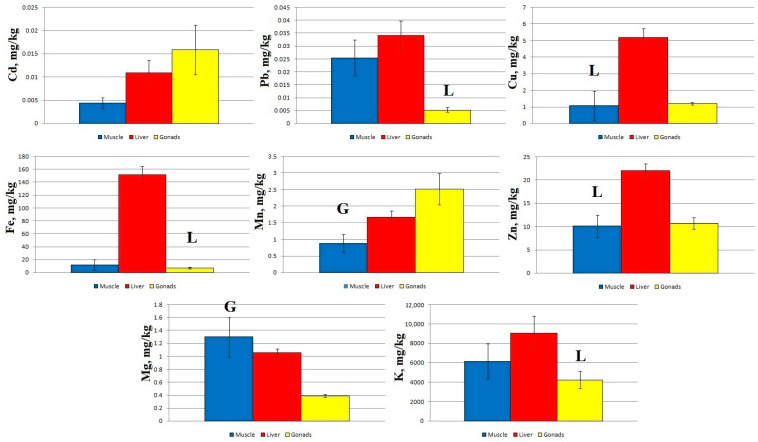
Concentration of micro- and macro-nutrients in different parts of sabrefish from Station 2 of the Rybinsk Reservoir. G—statistically significant differences from the gonads and muscles or liver, respectively. L—statistically significant differences from the liver and gonads or muscles, respectively. Muscles *n* = 10, liver *n* = 10, gonads *n* = 10.

At Station 2, statistically significant differences in Pb, Fe, and K concentrations were shown for the liver and gonads, in Cu and Zn concentrations for the muscles and liver, and in Mn and Mg concentrations for the muscles and gonads (Figure 3).

**Figure 4:**
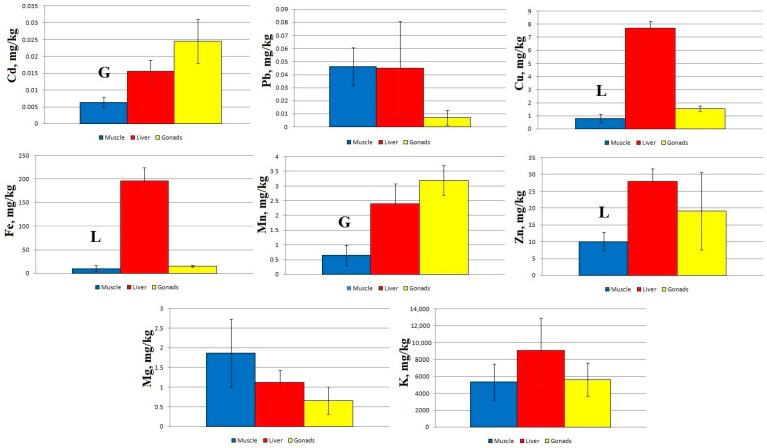
Concentration of micro- and macro-nutrients in different parts of sabrefish from Station 3 of the Rybinsk Reservoir. G—statistically significant differences from the gonads and muscles, respectively. L—statistically significant differences from the liver and gonads or muscles, respectively. Muscles *n* = 12, liver *n* = 12, gonads *n* = 10.

At Station 3, statistically significant differences are found between the concentrations of Cd and Mn in the muscles and gonads of sabrefish, as well as between the concentrations of Cu, Fe, and Zn in the muscles and liver (Figure 4).

**Figure 5:**
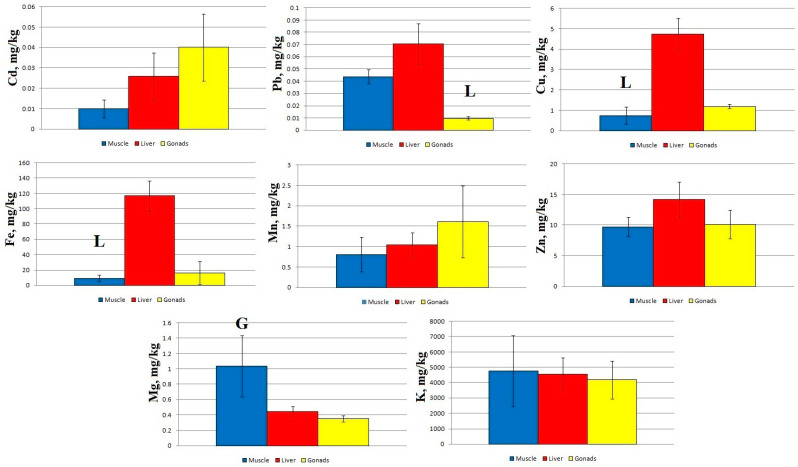
Concentration of micro- and macro-nutrients in different parts of sabrefish from Station 4 of the Rybinsk Reservoir. G—statistically significant differences from the gonads and muscles, respectively. L—statistically significant differences from the liver and gonads or muscles, respectively. Muscles *n* = 10, liver *n* = 10, gonads *n* = 9.

At Station 4, significant differences were found between the content of Pb in the liver and gonads, between the concentration of Cu and Fe in the muscles and liver, and between Mg concentrations in the muscles and gonads (Figure 5).

Regardless of the sampling area, the concentration of Cd and Mn increased in the following order: muscles → liver → gonads. Zn concentration at Stations 2–4 increased in the order muscles → gonads → liver, and at Station 1 it increased in the order muscles → liver → gonads. The other elements accumulated mainly in the liver of sabrefish, with the exception of Mg, which accumulated more intensively in the fish muscles at Stations 2–4.

### 3.3. Health Risk Assessment

The values of THQ and HI for specific tissues of sabrefish from different areas of the reservoir are presented in Table 3. The results of the study show that the THQ and HI indices for all metals do not exceed the permissible threshold (<1).

The HI value, regardless of the sampling area, decreases in the following order: liver → gonads → muscles.

The carcinogenic risk is calculated only for Cd and Pb because the carcinogenic potency slope factor of carcinogens (CSF) exists only for these metals (Table 4).

The calculated Pb values are less than 1 × 10^−6^ and Cd values range from 8.32 × 10^−6^ in the muscles at Station 1 to 1.22 × 10^−4^ in the gonads at Station 4 (Table 4).

## 4. Discussion

This study has revealed a tendency for more intensive accumulation of heavy metals (Cd, Pb, Zn, Cu, Mn, and Fe) in sabrefish from an area with a high anthropogenic load. A number of studies have also shown that stations located near the industrial complex (Stations 3 and 4) are considered the most unfavorable for aquatic organisms, in terms of heavy metal concentrations in both the water and bottom sediments and according to the results of biotesting in different areas of the reservoir [66,77,87,88].

In order to assess the degree of water pollution at the studied stations, the concentrations of heavy metals recorded in sabrefish during this study were compared with those from other studies. As mentioned above, only limited information is available on heavy metal concentrations in this fish species. Acomparison of our results with the data published by Subotić [55] suggests that in our study Cd, Cu, Fe, and Mn concentrations in the muscles and gonads of sabrefish are higher since they were obtained for wet weight and are comparable or exceed those in sabrefish from the Danube River, expressed on a dry weight basis. The heavy metal contents of Cd, Cu, Pb, and Zn in the muscles of sabrefish from the Rybinsk Reservoir are higher than from Lake Ladoga and lower than in this fish species from the Caspian Sea and water bodies of Moldova [52,53,54]. The literature data and the results of our study show that the metal concentrations in fish tissues vary widely depending on the sampling area. It is known that heavy metal contents in aquatic organisms are influenced not only by the anthropogenic load on the water body, the concentration of elements, and the duration of exposure, but also by the hydrochemical factors of the aquatic environment, because the solubility of a number of trace elements located in hard-to-reach compounds in silts depends on the oxygen level, pH, and other environmental parameters [89,90,91,92].

In our study, no single pattern of distribution of metals in the sabrefish body was found, with the exception of the liver—the organ responsible for the redistribution and detoxification of heavy metals [93,94,95]. In addition, the concentrations of metals in the liver are proportional to those present in the aquatic environment [96,97]. Probably due to this reason, in this organ, a general pattern of element accumulation was revealed for sabrefish from different stations of the reservoir, which may reflect the level of pollution of the entire reservoir.

In all the tissues of sabrefish, the content of elements such as K, Fe, and Zn significantly exceeded those of the other substances studied. At the same time, the concentration of K was several times higher than other elements. These metals are vital for living organisms and are called essential [98,99]. Normally, they should accumulate in large quantities because of their important role in the work of biological systems (enzymatic, metabolic, regulatory, and other roles) [100,101,102,103]. Deficiency of essential elements can lead to improper enzyme-mediated metabolic functions, congenital anomalies, immunological disorders, and chronic diseases [102,104,105,106,107,108]. High levels of K, Fe, and Zn in fish, including pelagic species, have also been reported by other researchers both in freshwater and marine ecosystems [101,104,109,110,111]. The highest K values, in comparison with other elements, were observed in the Black Sea kalkan *Psetta Maxima Maeotica* [108]. Potassium is one of the most important minerals in the body. It is involved in acid–base balance, glycogenesis reactions, regulation of osmotic pressure, conduction of nerve impulses, and muscle contractions [105,112].

In our study, the contents of Cd and Pb in the analyzed tissues of sabrefish were the lowest. Similar results, where the concentrations of essential elements exceeded those of Cd and Pb, were shown for both marine and freshwater pelagic fish species inhabiting rivers as well as lakes [96,113,114,115,116,117,118]. The reason for the low accumulation of nonessential metals (Cd and Pb) is the lack of need for them in physiological processes in living organisms [119]. Nevertheless, there are studies showing that the content of toxic elements in fish organs may exceed the concentration of essential ones due to the high level of anthropogenic load [120].

The pattern of macro- and micro-element distribution in the organs of sabrefish had some special features: an increased content of Cd and Mn was recorded in the gonads, an increased content of Mg was found in the muscle tissue, and other elements accumulated mainly in the liver of this fish species (Figure 2, Figure 3, Figure 4 and Figure 5).

Gonads are important reproductive organs responsible for producing gametes needed for fertilization [121]. In general, lower concentrations of heavy metals in the sex glands of fish may indicate a certain physiological mechanism of protection in these organs from the effects of heavy metals in order to avoid disruption of their work [122]. However, elevated Cd levels in the testes of sabrefish pose a risk for the reproduction of this species in the reservoir. Our results are consistent with findings of a number of studies on freshwater fish species, which have also reported higher Cd concentrations in the gonads in comparison with those in the liver and muscles [122,123,124]. In the gonads of sabrefish from the Danube River, Cd concentrations were below the detection limit [55].

Manganese is an essential microelement that acts as a cofactor in many enzymatic processes, and its deficiency can lead to reproductive abnormalities [102,125,126,127]. A higher Mn content in the gonads than in the muscles and liver of marine and freshwater fish species has been reported in a number of studies [33,123,128]. However, in the gonads of sabrefish from the Danube River, the content of Mn was lower compared to the muscles and liver [55].

It is known that magnesium ions are concentrated in the intercellular space of soft tissues and are in a bound form in fish muscles [129]. In the marine pelagic species *Caesio varilineata* and *Caesio lunaris*, the magnesium content is higher in the muscles than in the gonads and liver [130]. However, in Van fish (*Alburnus tarichi*), a cyprinid fish species which lives in the alkaline Lake Van, the Mg content in the muscles is lower than in the liver and gonads [131].

The liver is considered the main organ of metabolism and the most metabolically active tissue which accumulates and neutralizes toxic substances, including heavy metals [33,122,132]. It is known that the liver is a target organ for most metals, regardless of their route of entry, and is the optimum tissue for water monitoring, since higher concentrations of metals remain in this organ for a long time [96,97,115]. The results of our study regarding the greater accumulation of Fe and Cu in the liver of sabrefish are comparable to the work by Subotić [55] on a similar fish species. The literature data confirm that Pb accumulates more intensively in the liver of freshwater fish than in muscle tissue [92,133]. Despite the fact that muscles are not an active participant in the process of accumulation of the elements under study, they need to be analyzed, since they are considered the main edible part of fish and are important for human health [33,134].

It is interesting to note that in our study, the concentration of Zn at conditionally clean Station 1 was higher in the gonads, while at the other stations it was higher in the liver. It is known that this element plays an important role in the development of reproductive organs and fish reproduction, and its concentration in the gonads can be several times higher than in the muscles [108,132]. The tissues of sabrefish from Stations 2–4, subjected to anthropogenic load, contained higher concentrations of cadmium than the fish collected at Station 1. Zinc can be replaced by cadmium, reducing the harmful effects of the latter [135,136]. Perhaps for this reason, the zinc content in the gonads of fish from Station 1 was higher than in fish caught at the other stations.

Fish consumption is one of the main sources of heavy metal exposure to humans [84]. A human health risk assessment, associated with the duration of exposure to heavy metals, was performed using the recorded concentrations of Cd, Pb, Zn, Cu, Mn, and Fe in the tissues of sabrefish. The THQ data obtained in this study show that there is no potential risk for people related to the consumption of sabrefish from the Rybinsk Reservoir. The HI corresponded to the THQ model and did not exceed the permissible limit (<1). Thus, people will not experience non-carcinogenic health effects when consuming sabrefish from the Rybinsk Reservoir.

It is known that a number of toxic elements, such as lead, cadmium, methylmercury, and arsenic, have carcinogenic, mutagenic, and teratogenic effects when they enter the human body due to their insufficient excretion [86,137]. Carcinogenic risk (TR) indicates an increased likelihood of developing cancer in a person over the course of their lifetime due to the exposure to potential carcinogens [138]. The risk of developing cancer is considered insignificant, at TR < 1 × 10^−6^. When TR > 1 × 10^−4^, the consumers are in the unacceptable risk zone and some correction is needed [86,139,140].

In our study, the carcinogenic risks of Pb were less than 1 × 10^−6^, which indicates the absence of carcinogenic effects from sabrefish associated with this metal. As for Cd, theresults showed a slightly different picture. The consumption of muscle tissue of fish caught at Station 1 can be considered conditionally safe. For the other tissues, the risk of cancer from Cd is 1 per 100,000, and with regular lifetime consumption of fish gonads from Station 4 it is 1 per 10,000, because the TR values for these tissues exceeded 1 × 10^−4^. Long-term exposure to Cd can cause kidney failure, disrupt the gastrointestinal tract, reduce bone mineral density, and cause osteoporosis [102,104,117]. It is worth noting that in the areas adjacent to the reservoir, there is a steady increase in oncological diseases among all age groups of the population, and the highest number of cancer cases in the country [141,142].

Thus, due to potential human health risks associated with consumption of sabrefish, it is recommended to constantly monitor the levels of metals in their tissues, especially in fish caught from areas with an increased anthropogenic load. Given the target values of the carcinogenic risk for cadmium, we do not recommend using the internal organs of sabrefish from the Rybinsk Reservoir for food.

## 5. Conclusions

In this study, the concentrations of macro- and micro-elements in the muscles, liver, and gonads of sabrefish from Rybinsk Reservoir areas with different levels of anthropogenic load were determined. Heavy metals accumulated more intensively in the body of fish from more polluted areas of the reservoir. Among the analyzed elements, the maximum accumulation levels were found for K, Zn, and Fe and the minimum for Cd and Pb. The gonads contained the highest concentration of Cd and Mn, the muscles contained the highest concentration Mg, and the other elements accumulated mainly in the liver of sabrefish. In regard to human health, the THQ and HI values for all the elements did not exceed 1, which suggests that there is no potential non-carcinogenic risk to human health from heavy metals. The target values of carcinogenic risk (TR) for lead in all the tissues of fish from all the stations were below the threshold of 10^−6^, and for cadmium they ranged from 8.32 × 10^−6^ in the muscles of fish from Station 1 to 1.22 × 10^−4^ in the gonads of fish from Station 4. The increased content of cadmium in the gonads of sabrefish not only poses a risk to human health, but also to the reproduction of this species in the Rybinsk Reservoir. The data obtained in this study on the elemental content, including concentrations of toxic elements in the tissues of sabrefish, complement and expand our knowledge on the chemical and environmental situation in the Rybinsk Reservoir.

## Figures and Tables

**Table 1:** Dimensional and mass characteristics of sabrefish.

Sampling Station	*n*	Fish Length, cm	Fish Weight, g	Fish Weight without Entrails, g
1	7	32.7 ± 2.1	425 ± 85	368 ± 66
2	10	25.4 ± 0.8	175 ± 17	157 ± 16
3	12	23.7 ± 0.4	129 ± 7	117 ± 6
4	10	26.8 ± 1.2	208 ± 31	190 ± 29

**Table 2:** The content of micro- and macro-elements in the muscles, liver, and gonads of sabrefish.

Organs	Sampling Station	*n*	Cd	Pb	Zn	Cu	Mn	Fe	Mg	K
Muscles	1	7	0.003 ± 0.001 ^a^	0.016 ± 0.004	9.33 ± 1.72	1.15 ± 0.98	0.52 ± 0.13	8.49 ± 3.63	0.64 ± 0.03 ^a^	2628 ± 390 ^a^
	2	10	0.004 ± 0.001	0.025 ± 0.007	10.11 ± 2.42	1.08 ± 0.88	0.87 ± 0.26	12.05 ± 8.21	1.30 ± 0.31	6157 ± 826 ^b^
	3	12	0.006 ± 0.002	0.046 ± 0.014	10.00 ± 2.79	0.79 ± 0.32	0.65 ± 0.34	9.71 ± 6.45	1.86 ± 0.87 ^b^	5350 ± 2147
	4	10	0.010 ± 0.004 ^b^	0.044 ± 0.006	9.71 ± 1.62	0.75 ± 0.43	0.81 ± 0.42	9.13 ± 4.42	1.04 ± 0.40	4756 ± 2331
Liver	1	7	0.007 ± 0.002	0.022 ± 0.006	16.03 ± 2.22	4.03 ± 0.63 ^a^	1.19 ± 0.33	100.56 ± 57.54	0.58 ± 0.17	5570 ± 1798
	2	10	0.011 ± 0.003	0.034 ± 0.006	22.08 ± 1.48	5.17 ± 0.56	1.66 ± 0.19	151.25 ± 13.39	1.06 ± 0.06	9051 ± 1774
	3	12	0.016 ± 0.003	0.045 ± 0.036	27.95 ± 3.83 ^a^	7.70 ± 0.53 ^b^	2.40 ± 0.67 ^a^	196.22 ± 28.0	1.12 ± 0.30	9083 ± 3873
	4	10	0.026 ± 0.012	0.070 ± 0.017	14.17 ± 2.87 ^b^	4.73 ± 0.80	1.04 ± 0.31 ^b^	117.04 ± 19.81	0.44 ± 0.06	4563 ± 1056
Gonads	1	6	0.011 ± 0.003	0.003 ± 0.000	20.23 ± 4.50	0.31 ± 0.25 ^a^	1.87 ± 1.48	125.06 ± 39.26 ^a^	1.92 ± 0.75	11,224 ± 168
	2	10	0.016 ± 0.005	0.005 ± 0.001	10.63 ± 1.28	1.19 ± 0.09	2.51 ± 0.48	6.93 ± 1.01 ^b^	0.39 ± 0.02	5622 ± 1984
	3	10	0.024 ± 0.007	0.007 ± 0.006	19.11 ± 11.48	1.55 ± 0.21 ^b^	3.19 ± 0.51	15.38 ± 1.55	0.66 ± 0.34	4186 ± 1236
	4	9	0.040 ± 0.016	0.010 ± 0.001	10.10 ± 2.33	1.19 ± 0.10	1.61 ± 0.88	16.12 ± 14.72	0.35 ± 0.04	4249 ± 918

Note: small letters (^a^ > ^b^) denote statistically significant differences between stations in the same tissue samples.

**Table 3:** Target hazard quotient (THQ) and hazard index (HI) of sabrefish.

Organs	Sampling Station	*n*	THQ						HI
			Cd	Pb	Zn	Cu	Mn	Fe	
Muscles	1	7	0.001	0.001	0.006	0.006	0.001	0.002	0.017
	2	10	0.001	0.001	0.007	0.005	0.001	0.003	0.019
	3	12	0.001	0.002	0.007	0.004	0.001	0.003	0.018
	4	10	0.002	0.003	0.007	0.004	0.001	0.003	0.019
Liver	1	7	0.001	0.001	0.011	0.020	0.002	0.029	0.065
	2	10	0.002	0.002	0.015	0.026	0.002	0.044	0.091
	3	12	0.003	0.003	0.019	0.039	0.003	0.057	0.124
	4	10	0.005	0.004	0.010	0.024	0.002	0.034	0.078
Gonads	1	6	0.002	0.000	0.014	0.002	0.003	0.036	0.057
	2	10	0.003	0.000	0.007	0.006	0.004	0.002	0.022
	3	10	0.005	0.000	0.013	0.008	0.005	0.004	0.035
	4	9	0.008	0.001	0.007	0.006	0.002	0.005	0.029

**Table 4:** Target cancer risk (TR) estimates of sichel sampled.

Organs	Sampling Station	*n*	Cd	Pb
Muscles	1	7	8.32 × 10^−6^	2.77 × 10^−8^
	2	10	1.33 × 10^−5^	4.39 × 10^−8^
	3	12	1.94 × 10^−5^	6.76 × 10^−8^
	4	10	3.02 × 10^−5^	7.53 × 10^−8^
Liver	1	7	2.08 × 10^−5^	3.71 × 10^−8^
	2	10	3.30 × 10^−5^	5.88 × 10^−8^
	3	12	4.72 × 10^−5^	7.77 × 10^−8^
	4	10	7.84 × 10^−5^	1.21 × 10^−7^
Gonads	1	6	3.46 × 10^−5^	5.25 × 10^−9^
	2	10	4.83 × 10^−5^	9.26 × 10^−9^
	3	10	7.45 × 10^−5^	1.23 × 10^−8^
	4	9	1.22 × 10^−4^	1.70 × 10^−8^

## Data Availability

All data generated or analyzed during this study are included in this published article.
